# Hormonal treatments in metastatic endometrial stromal sarcomas: the 10-year experience of the sarcoma unit of Royal Marsden Hospital

**DOI:** 10.1186/s13569-015-0024-0

**Published:** 2015-03-15

**Authors:** Eirini Thanopoulou, Aleksandar Aleksic, Khin Thway, Komel Khabra, Ian Judson

**Affiliations:** Sarcoma Unit, Royal Marsden NHS Foundation Trust, Chelsea, London SW3 6JJ UK

**Keywords:** Endometrial stromal sarcoma, Aromatase inhibitors, Progestins, Hormonal treatment

## Abstract

**Background:**

Hormonal manipulation is sometimes recommended in the treatment of metastatic endometrial stromal sarcoma, but there are few data assessing the efficacy of endocrine therapies in this subtype of uterine sarcomas.

**Methods:**

We performed a retrospective electronic medical record review of patients with metastatic ESS treated with a hormonal agent at Royal Marsden Hospital between 1999 and 2011. We assessed progression-free survival (PFS), objective response and toxicity profile among patients with measurable disease.

**Results:**

Thirteen patients with metastatic ESS were treated with hormonal therapies. Hormone receptor status (estrogen and progesterone receptors) was assessed in 9 out of 13 patients and in all of them it was moderately to strongly positive. Aromatase inhibitors (AIs) were prescribed as first endocrine line in 11/13 patients and progestins in the remainder, while in 2^nd^ line treatment AIs were prescribed in 7/10 patients, followed by progestins and GnRH analogues. Median PFS for 1^st^line was 4.0 years (95% CI: 2.4 – 5.5 years) with 5-year progression-free rate of 30.8% (95% CI: 5.7 – 55.9%), both of which reflect the indolent natural history of ESS. Best objective response was partial response (PR) in 6/13 patients (46.2%; 95% CI: 19.2 – 74.9) and clinical benefit rate (defined as complete response + PR + stable disease ≥6 months) was 92.4% (95% CI: 64.0 – 99.8%; 12/13 patients). Median PFS for 2^nd^ line was 3.0 years (95% CI: 2.0 – 4.1 years) with 2-year progression-free rate of 88.9% (95% CI: 68.3 – 100.0).

**Conclusions:**

In this cohort of metastatic ESS patients, 1st line endocrine treatment achieved objective response in 46.2% of them and clinical benefit in 92.4%. Tamoxifen and hormone replacement therapy should not be prescribed in patients with ESS due to their detrimental effects. Until more solid data are available, a reasonable recommendation would be that 1^st^ line treatment with an endocrine treatment, preferably with an AI. Moreover, in view of the positive outcomes of our patients that received 2^nd^/3^rd^line endocrine treatments, all available hormonal options should be used in sequence in the management of ESS.

## Background

Endometrial stromal sarcoma (ESS) accounts for less than 10% of all uterine sarcomas, and is characterised by an indolent natural history, with a 5-year disease-specific survival of approximately 90% for stages I–II and 50% for advanced stages III–IV [1]. However, even in early stages an almost 50% recurrence rate has been documented [2]. All ESS are low grade according to current terminology [1].

The mainstay of treatment of localized ESS is total abdominal hysterectomy (TAH) with bilateral salpingo-oophorectomy (BSO) [1]. There is no established adjuvant treatment, though endocrine treatment has increasingly been recommended [1,3]. For women with recurrent advanced unresectable ESS, systemic hormonal treatment is recommended with palliative intent; several lines of endocrine treatment combined with interval cytoreductive surgery may significantly prolong overall survival [1]. However, when the disease has become resistant to estrogen deprivation, patients may be treated with chemotherapy, with less favourable outcomes [4,5].

ESS is well known to be hormonal receptor positive [1]; in the literature, estrogen receptor (ER) expression ranges between 40-100% [4,6,7] and progesterone receptor (PgR) between 60-100% [4,6,7]. As such, hormonal treatments resulting a reduction in estrogen drive have a leading role in therapeutic management, and appear to be more effective than chemotherapy [1,4]. Although the predictive role of ER and PgR has not been fully established in ESS yet, hormonal treatments have shown significant and durable responses in the metastatic setting and prolonged disease free survival in the adjuvant setting [1,3].

Due to the rarity of ESS, most of the data derive from case reports, with the bias that positive outcomes are more likely to be published [8-10] or small retrospective studies [4,6,7,11,12], which are characterised by small sample size with patients treated over a long period of time, both leading to marked heterogeneity in patient characteristics and interpretation of outcomes. Most of the data refer to progestins as they were first used to treat ESS, while currently aromatase inhibitors (AIs) are preferred as 1st line due to the higher therapeutic index, or as 2nd line in progestin-resistant disease [1]. With this in mind, we sought to record our institution’s experience in treating ESS patients with endocrine treatments.

## Methods

We performed a retrospective electronic medical record review of patients with advanced or recurrent ESS treated with hormonal manipulation on the Sarcoma Unit of the Royal Marsden Hospital (RMH) from January 1999 to July 2011. Patients were identified using the prospective Sarcoma Unit database and confirmed by pharmacy records. Patients were excluded if they had received any hormonal treatment to treat other diseases, such as breast cancer or had received concomitant chemotherapy. Patients’ electronic medical records were reviewed for age at diagnosis, stage, sites of metastases, volume of metastatic disease, tumour grade, hormone receptor status (ER and PgR), performance status, prior treatments, type and dose of hormonal agent used and toxicities. In addition, we recorded the presence or absence of co-morbidities.

All patients had surgical biopsies reviewed by the RMH department of pathology, which confirmed the diagnosis of ESS. Tumours were diagnosed as “low-grade” according to current terminology [13]*.* Immunohistochemistry for ER and PgR was performed on formalin fixed, paraffin embedded, representative, whole sections of tumour. Deparaffinized tumour sections were stained for ER and PgR (both supplied prediluted from Ventana Systems UK Ltd, Salisbury, UK) using heat-induced epitope retrieval. Appropriate positive and negative controls were used throughout.ER and PgR status was determined semiquantitatively and assigned as ‘weak’, ‘moderate’ or ‘strong’ in tumour nuclei.

The primary end-point of the analysis was progression-free survival (PFS), defined as time from the start of hormonal treatment until disease progression or death. Patients who had reached neither endpoint were censored at date of last follow-up. Hence, PFS1 was defined as time from the start of 1st line hormonal treatment until disease progression or death and PFS2 was defined as time from the start of 2^nd^ hormonal treatment until disease progression or death. The Kaplan–Meier method was used to estimate PFS. Objective response rate (ORR), defined as the rate of a complete response (CR) or partial response (PR) and clinical benefit rate (CBR), as the rate of CR, PR, and stable disease (SD) for at least 6 months wereevaluated by Response Evaluation Criteria in Solid Tumours (revised RECIST guideline, version 1.1) criteria [14]. Toxicity was graded using the National Cancer Institute Common Terminology Criteria for Adverse Events (CTCAE) version 4.02.

The study was approved by the Committee for Clinical Research of RMH.

## Results

### Patient and tumour characteristics

We identified 22 patients with locally recurrent and/or metastatic ESS treated with hormonal manipulation from January 1999 to December 2011. We excluded 9 cases that were either treated in another institution or presented as second opinion to our unit or which did not have sufficient clinical date for assessment. The demographics, tumour characteristics and prior surgical and radiotherapy treatment details of the remaining 13 patients are listed in Table 1. The median age at time of initiation of 1st line hormonal treatment was 49 years (range 39 to 70). All but one patient were postmenopausal at the time of 1^st^ line treatment (92%).

**Table 1:** **Patient and tumour characteristics (n = 13)**

**Variable (n = 13)**	**N (%)**
**Performance status (1** ^**st**^ ** line)**	
0	1 (7.7%)
1	12 (92.3%)
**Menopausal status**	
Premenopausal	1 (7.7%)
Postmenopausal	12 (92.3%)
**Number of comorbidities**	
0–1	11 (84.6%)
2–3	2 (15.4%)
**Initial management at diagnosis**	
Surgical resection alone	5 (38.5%)
Surgical resection and BSO	8 (61.5%)
**Prior exogenous estrogens**	
Tamoxifen	3 (23%)
HRT	7 (53.8%)
**Prior pelvic radiotherapy**	4 (30.8%)
**Sites of metastases at time of 1** ^**st**^ ** line**	
Pelvic	6 (46.2%)
Extrapelvic	3 (23%)
Both pelvic and extrapelvic disease	4 (30.8%)
**Tumour volume at 1** ^**st**^ ** line**	
Low	1 (7.7%)
High	12 (92.3%)
**Number of metastases at 1** ^**st**^ ** line**	
Oligometastatic	5 (38.5%)
Multiple	8 (61.5%)
**Hormone receptor status**	
**ER**	
Moderate to strong (grade 2–3)	9 (69.2%)
Weak (grade 1)	0 (7.1%)
NA	4 (30.8%)
**PgR**	
Moderate to strong (grade 2–3)	9 (69.2%)
Weak (grade 1)	0 (7.1%)
NA	4 (30.8%)

BSO, bilateral salphigoophorectomy; HRT, hormonal replacement therapy; ER, estrogen receptor; PgR, progesterone receptor NA, not available.

ER status was determined semi-quantitatively in 9/13 patients, and assigned as ‘weak’, ‘moderate’ or ‘strong’ in tumour nuclei. All nine patients had moderate to strong (grade 2–3) ER and PgR positive tumours.

All patients had measurable disease at time of initiation of 1st line hormonal treatment. All but one patient had high volume disease, which was defined as the presence of any tumour deposit >2 cm in longest diameter on radiographic imaging [15]. Moreover, 5 patients had oligometastatic disease defined as the presence of ≤5 metastatic deposits, while the other 8 patients had multiple metastases. Sites of disease included pelvis in 6 patients (46.2%), extrapelvic disease in 3 (23%) and both pelvic and extrapelvic disease (30.8%) in 4/13 patients (30.8%).

Seven patients (54%) received hormonal replacement therapy (HRT) either after surgery or to alleviate postmenopausal symptoms, with median duration of treatment 3.6 years (1.3 months −14 years; Table 1). Five of these patients were on HRT at the time of diagnosis of metastatic disease. One patient stopped HRT as initial hormonal manipulation and had partial response of her metastatic ESS that lasted 48 months; she was subsequently treated with letrozole and is still in remission (PFS 126 months). The other 4 patients stopped HRT and at the same time were started on 1st line endocrine treatment with an AI with subsequent regression of disease. Two out of the 13 patients were treated with tamoxifen at the time of diagnosis of metastatic ESS (duration of treatment 3 and 5 years respectively), and 1 patient was treated both with tamoxifen and HRT prior to developing metastatic ESS, further supporting previous findings [7]. All of them were started on 1st line treatment.

As shown in Table 2, non-steroidal AIs (NSAIs), namely letrozole 2.5 mg and anastrozole 1 mg daily, were given to 11/13 patients (84.6%), while 2/13 patients (15.4%) were treated with progestins. Of note, one of the patients was switched from megestrol acetate (MA) to letrozole because of allergic reaction to MA. Nine patients (69.3%) received hormonal manipulation as 1st line treatment without receiving any prior endocrine or chemotherapeutic agent prior to AI. Three patients were started on 1st line endocrine treatment as they progressed on 1st line chemotherapy, while the 4th patient was started on endocrine treatment (progestin) as maintenance treatment after achieving SD on 1st line chemotherapy.

**Table 2:** **Endocrine treatments details (n = 13)**

**1st line (n = 13)**	**N (%) median (range)**
**Median age at 1st line hormonal treatment**	49 years (39–70)
**Type of 1** ^**st**^ ** line hormonal treatment**	
NSAI	11 (84.6%)
Progestins	2 (15.4%)
**Response to 1** ^**st**^ **line**	
Complete response	0 (0%)
Partial response	6 (46.2%)
Stable disease (more than 6 months)	6(46.2%)
Progressive disease	1(7.6%)
**2nd line (n = 10)**	
Median age at 2^nd^ line hormonal treatment	59 years (43–72)
**Type of 2** ^**nd**^ **line hormonal treatment**	
NSAI	4 (40%)
Exemestane	3 (30%)
Progestins	2 (20%)
GnRH analogue	1 (10%)
**Response to 2** ^**nd**^ ** line**	
Complete response	0 (0%)
Partial response	0 (0%)
Stable disease (more than 6 months)	10(100%)
Progressive disease	0 (0%)

NSAI, non-steroidal aromatase inhibitor; GnRH, gonadotropin-releasing hormone.

In the 2^nd^ line setting (Table 2), the majority of patients were treated with AIs, either NSAIs (4/10 patients, 40%) or exemestane, an irreversible steroidal AI (3/10 patients, 30%). The other 2 patients received progestins and one patient (premenopausal) was treated with goserelin. All patients had progressed after 1^st^ line endocrine treatment and had measurable disease at time of 2nd line AI treatment; only one patient was lost in follow-up. Finally, 2 patients that eventually progressed on 2nd line endocrine treatment were treated with 3rd line of hormonal agents, specifically exemestane and letrozole.

### Objective responses to all lines of endocrine treatments

With a median follow-up of 7.8 years, the median duration of 1^st^ line endocrine treatment was 48 months (range 5–126 months). No patient achieved a CR, while PR was observed in 6 patients (46.2%), thus accounting for an ORR of 46.2% (95% CI: 19.2 – 74.9). All patients had high volume of disease, with only one of them having oligometastatic disease. The ER and PgR status was available in only 3/6 patients and in all 3 of them both were moderately to strongly positive. One patient is still on 1^st^ line endocrine treatment (94 months), but four patients eventually progressed and all of them were offered 2^nd^ line endocrine treatment, in view of the prolonged PFS and acceptable side effect profile. One patient was lost in follow-up.

Six patients (46.2%) had SD as best response, with all of them maintaining it for more than 6 months. All but one patient had high volume of disease, and 3/6 patients had oligometastatic disease. The ER and PgR status was available in 5 patients and in all of them expression of both was moderately to strongly positive. Of those patients that achieved SD, as best response, one patient is still on 1st line endocrine treatment (126 months), but the other five patients eventually progressed and all of them were offered 2^nd^ line endocrine treatment. Of note, one of these patients stopped her 1^st^ line treatment (letrozole) after 49 months, but her disease recurred 42 months later. Overall, the CBR (CR + PR + SD > 6 months) was 92.4% (95% CI: 64.0 – 99.8%). Finally, best response was progression of disease (PD) in one patient, who had high volume pelvic recurrence; hence she had debulking surgery after which she was started on 2^nd^ line endocrine therapy with medroxyprogesterone. Her disease was moderately ER and PgR positive.

The median duration of 2^nd^ line endocrine therapy was 21.6 months (range 11–47 months). All patients achieved SD as best response, with all of them maintaining it for more than 6 months. All but one patient had high volume of disease, and three of them had oligometastatic disease. The ER and PgR status was available in 7/10 patients and in all them both receptors’ status was moderately/ strongly positive. Overall, four of these patients are continuing their AI treatment to date (11–47 months), a patient progressed and has been referred for palliative care, two patients were switched to 3^rd^ line endocrine treatment, one was treated with sirolimus, one received 2 lines of chemotherapy, and one patient died with disease progression. With regards to the two patients that received 3^rd^ line endocrine treatment, one patient achieved SD for 10 months, while the other patient stopped treatment before full reassessment of her disease response.

### Progression-free survival

At last follow-up, 3 out of 13 patients (23%) had died with disease progression, 1 patient (7.7%) was alive with disease progression (palliative care), 2 (15.4%) were alive on 1st line letrozole without progression, 4 (30.8%) patients were alive on 2^nd^ line AI (letrozole and exemestane), 2 (15.4%) were alive on 3^rd^ line endocrine treatment and 1 patient was lost in follow-up.

**Figure 1:**
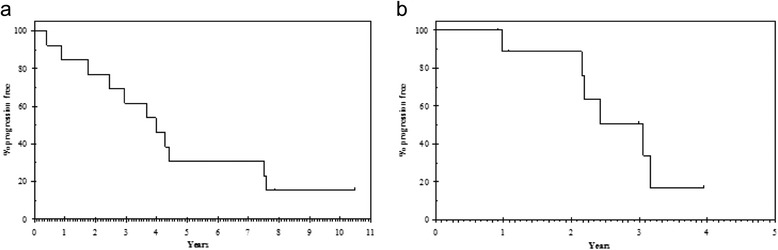
**Progression Free Survival (PFS).** In Figure 1 **a** is depicted the PFS defined as date of start of first line treatment to date of progression or death (PFS1), while in Figure 1 **b** is depicted the PFS defined as date of start of second line treatment to date of progression or death (PFS2). Surviving progression free patients are censored at last follow up.

Median PFS for 1^st^ line endocrine treatment (PFS1) was 4.0 years (95% CI:2.4 – 5.5 years; Figure 1a). The 2-year progression-free rate was 76.9% (95% CI:54.0 – 99.8%) and the 5-year progression-free rate 30.8% (95% CI:5.7 – 55.9%). Median PFS for 2^nd^endocrine treatment (PFS2) was 3.0 years (95% CI:2.0 – 4.1 years; Figure 1b). The 2-year progression-free rate was 88.9% (95% CI:68.3 – 100.0).

### Toxicities during aromatase inhibitor treatment - any dose reduction/ discontinuation

Toxicities observed during 1^st^ line treatment with AIs included only grade 1–2 side effects; specifically, grade 1 hot flushes in 2 patients, grade 2 arthralgias in one patient, grade 1 headache in one patient and grade 1 rash in another. The toxicity profile of progestins included only an allergic reaction to MA; hence the patient was switched to letrozole.

Similarly in the 2^nd^ line setting, only grade 1 hot flushes and joint pain was recorded in 1 out of 7 patients on AIs, while in the 3^rd^ line the main toxicities reported were osteoporosis in one patient that was started on supplementation treatment and grade 2–3 mood changes in another patient. With regards to progestins (MA), one patient developed significant weight gain and eventually stopped treatment.

## Discussion

Endometrial stromal sarcoma (ESS) is considered a hormone-dependent malignancy, however due to its rarity there are no prospective randomised studies on hormonal agents, data consisting instead of case reports and small retrospective series [1,16]. The rationale for using hormonal treatments in ESS is generally empirical, in view of the hormone receptor expression and low grade; hence, the hormonal strategies are mainly extrapolated from experience in treating hormone-positive breast cancer, which is much more common [1]. Progestins were among the first hormonal modulators to be explored in the management of ESS, followed eventually by 3^rd^ generation AIs, which are currently the preferred hormonal agents for 1st line treatment or 2nd line treatment in progestin-resistant ESS [1].

This case series adds to the current knowledge of the role of hormonal agents in the management of patients with ESS. In our cohort of 13 patients, with measurable advanced ESS, objective response was observed in 46.2% (all patients had PRs and were on AIs), and clinical benefit rate in 92.4% of patients. The only patient that progressed was moderately ER and PgR positive with high volume of disease. This high response rate is in accordance with published data [1,4,16] on 1st line treatment of ESS with progestins [4,16], and add to the sparse data on 1st line treatment with AIs [1]. Median PFS (PFS1) was 4.0 years (95% CI:2.4 – 5.5 years) with 5-year progression-free rate of 30.8% (95% CI:5.7 – 55.9%), both of which reflect efficacy of estrogen deprivation and the indolent natural history of ESS.

To our knowledge, this is the first case series of advanced ESS reporting outcomes of 2nd line endocrine treatment. In our cohort of 10 patients, all but one of which had a high volume of metastatic disease, no objective response was observed. However, all patients exhibited stable disease for more than 6 months. Median PFS (PFS2) was 3.0 years (95% CI:2.0 – 4.1 years) with 2-year progression-free rate of 88.9% (95% CI:68.3 – 100.0). Moreover, we report for the first time two patients with metastatic ESS that were treated with 3rd line endocrine treatment. Hence, the optimal treatment strategy in advanced ESS would appear to be cytoreductive surgery followed by sequential lines of hormonal agents [1,6,17].

As expected, all hormonal treatments had a favourable toxicity profile with mainly grade 1–2 side effects. Notable is an allergic reaction to MA, for which the patient was switched to letrozole and significant weight gain on MA by another patient, who eventually stopped treatment. Moreover, one patient on exemestane developed osteoporosis and was started on bisphosphonates.

Finally, the detrimental effect of HRT and tamoxifen has been shown in this cohort, as 7/13 patients received prolonged HRT (duration of HRT 6–168 months) and 3/13 patients were treated with tamoxifen at the time of diagnosis of metastatic ESS; disease responses were observed with withdrawal of these treatments, further supporting previous findings [6,7]. The 2014 update of NCCN and ESMO guidelines have incorporated the contraindication of tamoxifen and HRT in patients with ESS [18,19].

The main limitations of this study are its retrospective nature and the small number of patients, which reflect the rarity of this subset of uterine sarcomas. Hence, the results should be interpreted cautiously. Most literature data to date refer to the use of progestins as 1^st^ line endocrine treatment with Cheng et al. showing in the largest cohort (30 patients) that hormonal treatment was more effective than radiotherapy and chemotherapy [4]. Until prospective studies provide mature data, it is reasonable to recommend that the 1^st^ line treatment of ESS should comprise an endocrine agent, preferably an AI [1,19].

## Conclusions

ESS is clearly a hormone sensitive subtype of uterine sarcomas with an indolent natural history and significant clinical responses to endocrine manipulation [1,4,11,19]. Thus, all available hormonal options should be used in sequence for the management of ESS. Moreover, the adverse effects of tamoxifen and HRT have been highlighted and their use is contraindicated in patients diagnosed with ESS [1,6,7]. Our cohort adds to the limited data available for the management of ESS [1]. It remains to be clarified if the favourable outcomes shown in our study are driven predominantly by the favourable biology of ESS, and to quantify any correlation between hormonal receptor expression and response to endocrine treatment, similar to that seen in hormone positive breast cancer [1,11].

## Abbreviations

AIs: Aromatase inhibitors
BSO: Bilateral salpingo-oophorectomy
CB: Clinical benefit
CBR: Clinical beniefit rate (CR + PR + SD ≥ 6 months)
CR: Complete response
CTCAE: Common Terminology Criteria for Adverse Events
ER: Oestrogen receptor
ESS: Endometrial stromal sarcoma
HRT: Hormonal replacement therapy
MA: Megestrole acetate
NSAI: Non-steroidal AI
ORR: Objective response rate
PR: Partial response
PgR: Progesterone receptor
PD: Progression of disease
PFS: Progression-free survival
RECIST: Response Evaluation Criteria in Solid Tumours
RMH: Royal Marsden Hospital
SD: Stable disease
TAH: Total abdominal hysterectomy
